# Novel NtA and LG1 Mutations in Agrin in a Single Patient Causes Congenital Myasthenic Syndrome

**DOI:** 10.3389/fneur.2020.00239

**Published:** 2020-04-09

**Authors:** Aiping Wang, Yangyang Xiao, Peng Huang, Lingjuan Liu, Jie Xiong, Jian Li, Ding'an Mao, Liqun Liu

**Affiliations:** ^1^Department of Pediatrics, The Second Xiangya Hospital, Central South University, Changsha, China; ^2^Department of Pediatrics Neurology, Children's Medical Center, The Second Xiangya Hospital, Central South University, Changsha, China

**Keywords:** *AGRN*, agrin, congenital myasthenic syndrome, whole-exome sequencing, missense mutation

## Abstract

Congenital myasthenic syndrome (CMS) is a group of genetic disorders of neuromuscular transmission that is characterized by muscle weakness. A mutation in the gene encoding agrin (*AGRN*) is a rare cause of CMS, and only a few families or isolated cases have been reported. We reported a pediatric proband exhibiting muscle weakness in the trunk and limbs with skeletal malformation and intellectual disability and performed whole-exome sequencing (WES) of the proband parent-offspring trio. Results revealed a new compound heterozygous mutation in *AGRN*: c.125A>C (p.Glu42Ala) in the N-terminal agrin domain (NtA) and c.4516G>A (p.Ala1506Thr) in the laminin G1 domain (LG1). Bioinformatic analysis predicted the mutation as possibly pathogenic. The new compound heterozygous mutation in *AGRN* may disrupt agrin's known function of bridging laminin and α-dystroglycan and undermine the formation and maintenance of the neuromuscular junction (NMJ) via both muscular and neural agrin pathways. It may also induce secondary peripheral neuropathy and skeletal malformation.

## Introduction

As a diverse group of genetic disorders with an onset at birth or in early childhood that affects neuromuscular transmission, congenital myasthenic syndrome (CMS) is characterized by muscle weakness of limb, axial, facial, ocular, and/or bulbar muscles. Aside from clinical findings, characteristic electromyography (EMG) of CMS shows a decremental response of the compound muscle action potential (CMAP) toward low-frequency stimulation (2–3 Hz). CMS is also characterized by normal or slightly elevated serum creatine kinase (CK) concentrations, an absence of anti-muscle-specific tyrosine kinase (MuSK) and anti-acetylcholine receptor (AChR) antibodies in the serum, a positive response to acetylcholinesterase (AchE) inhibitors, and a lack of improvement following immunosuppressive therapy. Muscle biopsies from patients with CMS have revealed no major abnormalities but have indicated a predominance of type-I fibers and occasional minor myopathic changes via routine immunohistochemical analysis. (1, 2). Currently, over 30 genes have been found to be related to CMS (3, 4). The gene encoding agrin (*AGRN*) is localized at chromosome 1p36.33 (5) and its mutation is one of the rarest causes of CMS, which accounts for 0.84% of all CMS cases and causes type-8 CMS (2). At present, only a few families or isolated cases have been reported with autosomal recessive *AGRN* mutations (Supplementary Table 1) (6–11). Agrin is an important heparin sulfate proteoglycan widely distributed throughout the body. It has many isomers owing to alternative mRNA splicing and amino acid insertions. Most isomers are secreted types that are expressed in both neural and non-neural tissues and binding to basal laminae (BL) components (Supplementary Figure 1) (12–14). Neural agrin (Z+ agrin), secreted by the axons of motor neurons into the NMJ cleft, has 8–19 amino acids inserted at the Z site. This insertion multiplies the capacity of binding to low-density lipoprotein receptor-related protein 4 (LRP4), activates the postsynaptic LRP4-MuSK complex and finally induces postsynaptic accumulation of acetylcholine receptors (AChRs) (15–17). Mutations in *AGRN* lead to agrin dysfunction, thereby affecting NMJ formation and maintenance, resulting in type-8 CMS (18, 19). In the present study, we found one pediatric case of CMS caused by a novel compound heterozygous mutation in the *AGRN* gene. This finding broadens our understanding of the clinical phenotypes of CMS and the mutational spectrum related to the *AGRN* gene.

## Case Presentation

The proband was a 5 year old girl suffering from muscle weakness soon after birth. She was the first child of a healthy non-consanguineous couple and was vaginally delivered at full-term with normal weight and Apgar scores. She was found to have ptosis of both eyelids soon after birth, rarely showed limb movements, and exhibited weakness in chewing and swallowing. She was unable to erect her head until she was 6 months old and was unable to crawl until she was 10 months old. She was unable to sit until she was 1.5 years old and has never been able to stand, even at the conclusion of the present study. She was unable to bilaterally move her upper arms or hold objects steadily in both hands. She had retardation of her language development; she started babbling at 1.2 years old and, at the time of the present study, was only able to speak at a low rate and with poor articulation. A former gene panel test showed negative results for spinal muscular atrophy and peroneal muscular atrophy.

**Figure 1:**
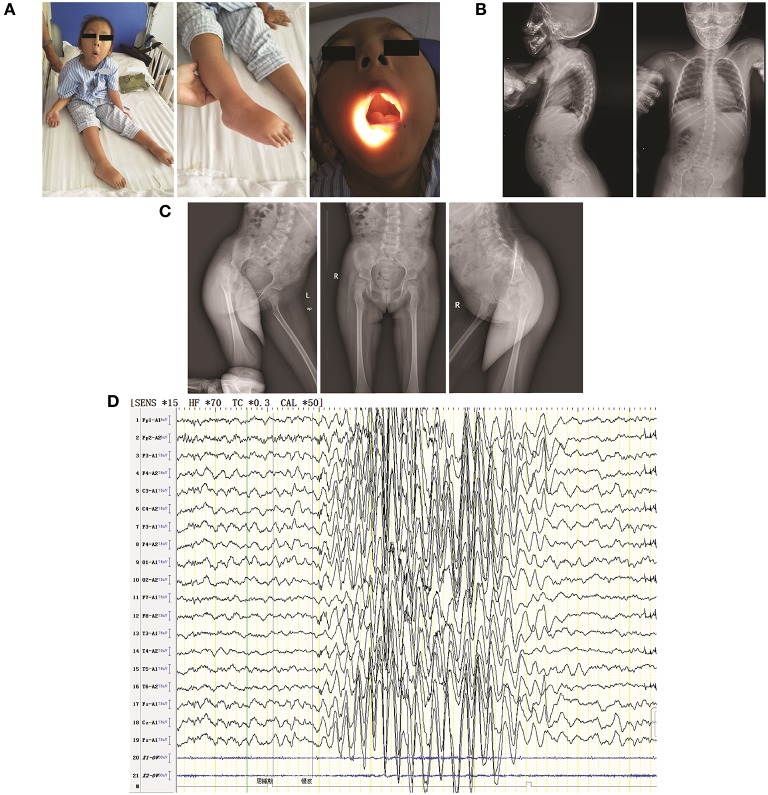
Clinical features of the patient in the present study. **(A)** The following visible symptoms are shown: bilateral ptosis (not shown due to censuring patient identity) with hyperextension of ankle and carpal joints (left); foot drop (middle); high-arched palate (right); **(B)** thoracolumbar scoliosis; **(C)** mild shallowness and bluntness of the right acetabular bone, indicating dysplasia; **(D)** EEG showing extensive 3–4.5 HZ. θ and δ waves mixed with non-sustained discharges of a small amount of low-amplitude spike/sharp waves during shallow sleep.

Physical examination confirmed the following: physical retardation (height, 97 cm; body weight, 16 kg); bilateral ptosis; hyperextension of ankle and carpal joints; foot dropping; amyotrophy in bilateral proximal lower limbs; hypotonia in all four limbs; no elicited tendon reflexes; low muscle strength [Medical Research Council (MRC) scale grade 3 in cervical muscle, grade 2 in bilateral proximal upper limbs, grade 3 in distal upper limbs, grade 1 in bilateral proximal lower limbs, and grade 2 in distal lower limbs]; normal sensation, and normal cutaneous plantar reflex. She also had a high-arched palate, enamel hypoplasia, and a small jaw; she did not exhibit nystagmus (Figure 1A). Thoracolumbar scoliosis and right acetabular dysplasia were revealed by X ray (Figures 1B,C). Her serum CK level (118.9 U/L) was normal and she was negative for anti-AChR and anti-MuSK antibodies. Her neostigmine test showed a negative result. Her EMG (Supplementary Tables 2–5) presented spontaneous potentials (in the form of positive sharp waves and fibrillations) as well as a reduction in motor unit recruitment for skeletal muscles of the limbs. Her motor unit potential (MUP) revealed an increased time course (14.2 ms of left extensoris digitorum communis and 14.4 ms of right tibialis anterior) but a normal amplitude. The conduction velocities of both her sensory and motor nerves were decreased. The amplitudes of both CMAP and sensory nerve action potential (SNAP) were decreased, whereas their peak latencies were prolonged. H-reflex waveforms were not elicited. Unfortunately, the patient did not cooperate with a repeated nerve stimulation examination. Electroencephalography (EEG) showed extensive 3–4.5 Hz, θ and δ waves mixed with non-sustained discharges of a small amount of low-amplitude spike/sharp waves during shallow sleep (Figure 1D). Assessment via the Wechsler Intelligence Scale revealed a low verbal intelligence quotient of 52, whereas the intelligence quotient could not be determined due to the patient's inability to perform bilateral hand movements. No abnormalities were found via blood-urinary metabolic screening, electrocardiography, visual/auditory evoked potentials, or magnetic resonance imaging of the head and spinal cord. The patient's parents refused muscle biopsies to further confirm the patient's diagnosis. To identify the ultimate cause, whole-exome sequencing (WES) was performed. It was approved by the ethics committee of the Second Xiangya Hospital of Central South University (approval No.: XY-LL20180408), and informed consent was obtained from the patient's parents.

## Gene Sequencing

Genomic DNA from blood was extracted to perform WES in the proband-parent trio. With an average sequence read depth of 100×, we captured targeted genes using xGen Lockdown Probes and Reagents (Integrated DNA Technologies, Inc., Coralville, IA, USA) according to the protocol of the manufacturer (Illumina). The captured libraries were sequenced by an IlluminaNextSeq 500. We aligned the sequence reads to the human reference genome (hg19) by utilizing a Burrows-Wheeler Aligner 0.6.1.11. Indels and single-nucleotide-polymorphisms (SNPs) were detected by the Genome Analysis Toolkit 1.6.7, SAMtools 0.1.18, and Picard 1.60. Variants were ultimately annotated with custom scripts. Genotypes were filtered to retain indels and SNPs with Phred-like quality scores at a minimum of 30. We primarily focused on variants that altered canonical splice sites and protein-coding regions. Variants with frequencies <1% in the following public databases were included: the Human Gene Mutation Database (HGMD), 1,000 Genomes, and the Single Nucleotide Polymorphism Database (dbSNP). Detected mutations were verified by Sanger sequencing in all three participants. Copy number variation (CNV) analysis was performed via CODEX, XHMM (v1.0), and KSCNV.

## Results

**Figure 2:**
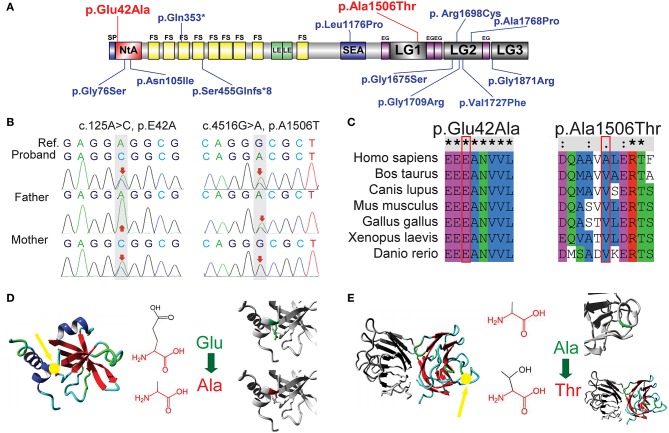
Sequence analysis of two novel AGRN mutations. **(A)** Previously reported mutations in AGRN of CMS cases are highlighted in blue, whereas the two reported AGRN mutations of the present study are highlighted in red. Note that most genetic mutations are located in NtA, LG2, and LG3 domains. **(B)** Trio analysis (i.e., patient and patient's parents) of novel mutations in the present study. The proband c.125A>C (p.Glu42Ala) is from the asymptomatic mother with a heterozygous mutation, whereas the proband c.4516G>A (p.Ala1506Thr) is derived from the asymptomatic father with a heterozygous mutation. The reference (Ref) derives from a cDNA sequence from GenBank. **(C)** Sequence conservation analysis. c.125A>C (p.Glu42Ala) is located in an extremely conserved fragment, but c.4516G>A (p.Ala1506Thr) was not as strictly conserved as c.125A>C across species. **(D)** Protein structural-effects analysis of c.125A>C (p.Glu42Ala) via HOPE. The change to a neutrally charged alanine (highlighted in red) from a negatively charged residue of glutamic acid (highlighted in green) disturbs the formation of a salt bridge and abolishes its function. The location of p42 is indicted by a yellow arrow. **(E)** Protein structural-effects analysis of c.4516G>A (p.Ala1506Thr). A smaller and less hydrophobic mutant threonine (highlighted in red) residue might disturb the core structure of the LG1 domain. The location of p1506 is indicted by a yellow arrow. EG, EGF-like domain; FS, follistatin-like domain; LE, laminin EGF-like domain; LG1/ LG2/ LG3/, the first/second/third laminin G-like domain; NtA, N-terminal agrin domain; SEA, sea urchin sperm protein, enterokinase, and agrin domain.

A novel heterozygous missense mutation in *AGRN* was identified from the patient: c.125A>C (p.Glu42Ala) in exon 1, inherited from her asymptomatic mother (heterozygous c.125A>C), and c.4516G>A (p.Ala1506Thr) in exon 26, inherited from her asymptomatic father (heterozygous c.4516G>A; Figures 2A,B). Using PolyPhen-2 and SNP&GO, a probable deleterious effect of c.125A>C on protein function was indicated (Score 0.974). Additionally, according to HOPE protein structural-effects analysis of *AGRN* (http://www.cmbi.ru.nl/hope/), the wild-type negatively charged residue of glutamic acid at position 42 forms a salt bridge with arginine at position 34, arginine at position 38, and arginine at position 39. These amino acids were located in an extremely conserved fragment (Figure 2C) in the important N-terminal agrin domain (NtA), and the mutation introduced a neutrally charged alanine, which may disturb this domain and abolish its function (Figure 2D). According to the American College of Medical Genetics and Genomics (ACMG) standard, we classified this mutation as likely pathogenic. In contrast, the other identified *AGRN* mutation, c.4516G>A, was interpreted by PolyPhen-2 to be a benign mutation because the wild-type residue at this position was not conserved (Figure 2C). However, the residue at position 1506 was located in the core of the first laminin G1 domain (LG1) and the mutation introduced a smaller and less hydrophobic mutant threonine residue. This mutated residue might disturb the core structure of this domain (Figure 2E), thus this mutation was interpreted as disease-related by SNP&GO.

According to the clinical manifestation and laboratory examination results, the patient was treated with pyridostigmine bromide (15 mg) once every 8 h. Muscle tone was improved only to a limited extent. Her parents refused salbutamol treatment when gene test showed possible pathogenic *AGRN* mutations. Unfortunately, we lost her follow-up 3 months later.

## Discussion

In the present study, a new compound heterozygous mutation in *AGRN*, c.125A>C in exon 1 (p.Glu42Ala), and c.4516G>A in exon 26 (p.Ala1506Thr), was identified in a pediatric patient with CMS. The clinical features of this patient were consistent with previous reports of CMS caused by *AGRN* (Supplementary Table 1 and Figure 2A) (6–11).

Neural agrin (Z+ agrin) plays a critical role in AChR aggregation because of the amino acids inserted at the Z site, which multiplies the capacity to bind to LRP4 (16, 17). However, another isomer could also participate in this process. Muscle agrin, lacking amino acid insertion at the Z site, is also critical for the formation and stabilization of the postsynaptic apparatus as a bridge for binding to laminin and α-dystroglycan (20, 21), although it has little binding capacity with LRP4 compared to that of neural agrin (16). The p.Glu42 and p.Ala1506 residues exist in both neural agrin and muscle agrin (Supplementary Figure 1).

The NtA, where p.Glu42 is located in agrin, binds to the synaptic basal lamina component, laminin, to provide the basis of agrin localization for AChR clustering (22). Major primary (α-helix 3) and secondary binding sites for agrin-laminin binding exist in the NtA, and Arg43 and Arg40 constitute the open face of the secondary binding site. It has been reported that a mutation of Arg43Ala decreases the agrin-laminin binding ability to 1/40 of that of wild type, and a triple combination of Glu23Ala, Glu24Ala, and Arg40Ala mutations decrease this binding ability to 1/20 of that of wild type (22, 23). We speculated that p.Glu42Ala may also be pathogenic for the following two reasons: (1) p42 is close to p40 and p43; and (2) the charged residues of arginine and glutamic acid are substituted by a neutrally charged hydrophobic alanine and may disturb the formation of a salt bridge.

**Figure 3:**
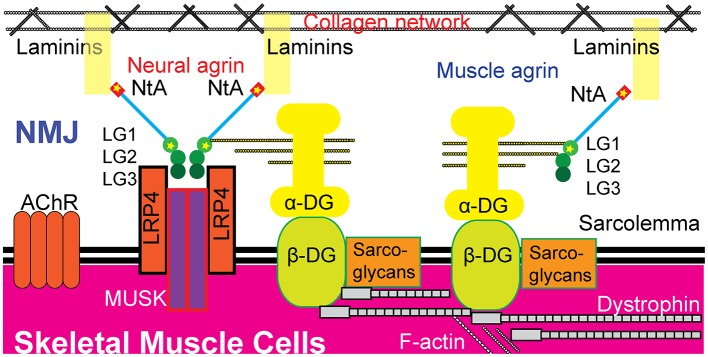
Possible pathogenic mechanisms of the two novel heterozygous missense mutations in AGRN. The p.Glu42 and p.Ala1506 residues exist in both neural agrin and muscle agrin. The p.Glu42Ala mutation was located in the NtA domain, which binds to laminin in the BL. The p.Ala1506Thr mutation was located in the LG1 domain, which binds to α-dystroglycan (α-DG). Neural agrin forms an agrin-LRP4 binary complex during AChR aggregation and LG1 has no direct role during this process. Whether or not p.Ala1506Thr interferes with AChR aggregation is unknown. Additionally, muscle agrin may act as a collateral linker by binding to the coiled-coil of laminins and to α-dystroglycan to function critically in the maintenance of the NMJ. Hence, these two mutations are hypothesized to potentially disrupt the formation and maintenance of the NMJ through both neural and muscle agrin pathways, leading to CMS. AChR, acetylcholine receptor; DG, dystroglycan; LG, laminin G-like domain; LRP4, low-density lipoprotein receptor-related protein 4; MUSK, muscle-specific kinase; NtA, N-terminal agrin domain; NMJ, neuromuscular junction.

Both muscle agrin and neural agrin have been shown to bind to α-dystroglycan through the LGs in the C-terminal (24–26). Agrin contains three LGs: LG1, LG2, and LG3. LG1 is the site of p.Ala1506Thr. Previous studies have shown that *AGRN* mutations in LG domains are concentrated in LG2 and LG3 (6–11). Neural agrin acts differently from other tissue-specific agrins; during NMJ formation, two neural agrins form an agrin-agrin dimer that then forms an agrin–LRP4 binary complex to active MUSK through the interface constituted by LG2 and LG3 (27). Additionally, a previous study showed that blocking α-dystroglycan binding to neural agrin failed to block AChR clustering (24). Hence, a p.Ala1506Thr mutation, which introduces a smaller and less hydrophobic threonine residue, might disturb the core structure of LG1 and possibly induce damage through muscle agrin's stabilization of the postsynaptic apparatus (20). Taken together, we hypothesize that the two mutations discovered in the present study, p.Glu42Ala and p.Ala1506Thr, damage the formation and maintenance of the NMJ through both neural and muscle agrin pathways, leading to CMS (Figure 3).

Although the patient's clinical signs and symptoms were consistent with CMS, her EMG showed a mixture of demyelination and axonal injury of peripheral motor and sensory nerves, which is not consistent with typical CMS EMGs. A previous study showed that the EMGs of a few CMS patients exhibited predominantly neurogenic changes (28). Additionally, in young children with NMJ disorder or myopathy, similar EMG results of neurogenic changes occur more commonly (29). This phenomenon may be attributed to myopathy or NMJ disease triggering motor neuron degeneration, which is a hypothesis that was partly supported in a transgenic mouse experiment (30). In this experiment, mice expressing mutant forms of the human superoxide dismutase-1 gene (hSOD1) only in skeletal muscle developed severe pathology, affecting myofiber and NMJ, and inducing secondary distal axonopathy of motor neurons. Muscle biopsies of CMS patients with *AGRN* mutations have revealed denervated and remodeled NMJs (10), which suggest the existence of secondary changes to motor nerves. On the other side of the NMJ, mutant agrin also influences the repair of peripheral nerves and remyelination. It has been shown that Schwann cells (SC) also secreted agrin, which then binds to laminin-2 and α-dystroglycan (31). Additionally, SC-specific agrin binding with glial cell line derived neurotrophic factor (GDNF), glial cell line–derived neurotrophic factor receptor α1 (GFRα1), and neural cell adhesion molecules (NCAM) within the lipid rafts of SCs initiates intracellular signal transduction and then activates the extracellular signal-regulated kinase 1/2 (ERK1/2) pathway, which provides a positive feedback loop for GDNF signaling in SCs. Disruption of this process impairs peripheral nerve regeneration and remyelination (32). In summary, we hypothesize that p.Glu42Ala and p.Ala1506Thr mutations may affect the formation of the NMJ via neural agrin, affect the maintenance of the NMJ via muscle agrin, and aggregate secondary peripheral neuropathy via SC agrin.

As a protein that is widely expressed throughout the body, agrin mutations may cause dysfunctions of other organ systems aside from the specific neuromuscular system (i.e., the NMJ) primarily discussed in the present study. The patient in the present study suffered from thoracolumbar scoliosis and acetabular dysplasia, which is consistent with a previous report that found skeletal malformations in CMS cases with *AGRN* mutations (10). It has been shown that chondrocyte-secreted agrin (splice variant of agrin [y0, z0]), which is another secreted isomer that binds with LRP4 and α-dystroglycan, is important in cartilage differentiation (33). It is possible that the two *AGRN* mutations that we found in the present study may also induce pathogenic effects of chondrocyte agrin. A noteworthy phenomenon of the patient in our present study was that she exhibited both intellectual disability and EEG abnormalities. Interestingly, a previous study has suggested an important role for agrin in central nervous system (CNS) development (34), and agrin is able to maintain blood-brain barrier (35). It has also been shown that the C-terminal of agrin can specifically bind to the α3 subunit of Na/K-ATPases to regulate the electrical activity of neurons (36). Hence, these findings collectively provide a strong rationale for future studies to determine if the newly found *AGRN* mutations in our present study are pathogenic in the CNS.

The present study had the following limitations: (1) a muscle biopsy for diagnosis was refused by the parents; (2) the patient did not cooperate with low-frequency stimulation during EMG testing; and (3) intronic mutations were not taken into consideration due to limitations in our genetic testing methods. Therefore, functional characterization of the consequences of p.Glu42Ala and p.Ala1506Thr mutations in *AGRN* will be required in future studies to determine the contributions of these mutations in SCs, chondrocytes, the NMJ, and the CNS.

## Conclusion

Our study revealed a new compound heterozygous mutation in *AGRN*, which may disrupt the known function of agrin of bridging laminin and α-dystroglycan, thereby undermining the formation and maintenance of the NMJ via both muscular and neural agrin pathways. This new mutation may also induce secondary peripheral neuropathy and skeletal malformation. Therefore, future studies will be required on the direct pathogenic mechanisms of the novel mutation in SC, chondrocytes, the NMJ, and the brain.

## Data Availability Statement

Publicly available datasets were analyzed in this study. This data can be found here: http://www.hgmd.cf.ac.uk/ac/index.php, https://www.internationalgenome.org/data/, and https://www.ncbi.nlm.nih.gov › snp.

## Ethics Statement

Written informed consent was obtained from the individual(s) and minor(s) legal guardian/next of kin, for the publication of any potentially identifiable images or data included in this article.

## Author Contributions

AW and YX conceived the study and participated in its design. AW, PH, and LinL provided medical care for the patients and collected data. AW, PH, YX, DM, and LiqL performed genetic analysis. AW and YX wrote the manuscript. YX, JL, and JX produced schematic figures. All authors read and approved the final manuscript.

### Conflict of Interest

The authors declare that the research was conducted in the absence of any commercial or financial relationships that could be construed as a potential conflict of interest.

## Abbreviations

AChR: acetylcholine receptor
CMS: congenital myasthenic syndrome
DG: dystroglycan
EMG: electromyography
LG: laminin G-like domain
LRP4: low-density lipoprotein receptorrelated protein 4
MuSK: muscle-specific tyrosine kinase
NMJ: neuromuscular junction
NtA: N-terminal agrin domain.
