# Supplementary material for: Novel NtA and LG1 Mutations in Agrin in a Single Patient Causes Congenital Myasthenic Syndrome
Source: Front Neurol. 2020 Apr 9;11:239. doi: 10.3389/fneur.2020.00239 (PMC7160337; doi:10.3389/fneur.2020.00239)
Supplement: Supplementary file 1 [file Table_1.DOCX]

Supplementary Material

## Supplementary Figure


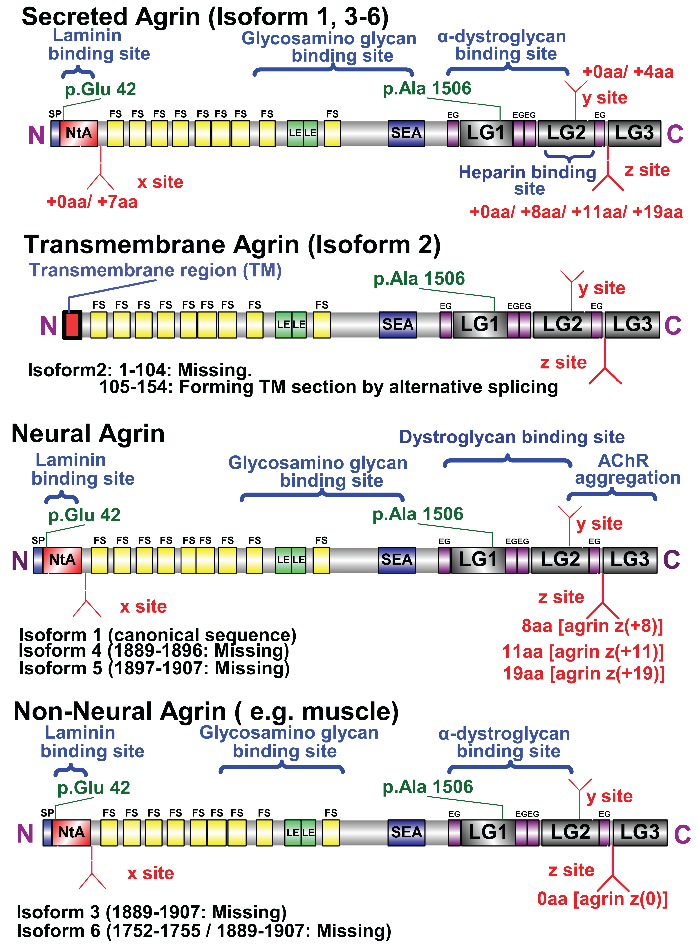


**Supplementary Figure 1.** Agrin isomers created by alternative mRNA splicing and amino acid insertions in X, Y, and Z sites. Isoform 1 is the full-length agrin. Most isomers are secreted types into basal laminae. Only one transmembrane type (isoform 2) exists and is mainly located in the plasma membrane of neurons. Motor neurons secrete neural agrin (isoforms 1, 4, and 5) with 8–19 amino acids inserted at the Z site (agrin [Z+]), which increases the capacity to bind to LRP4 and to play a critical role in AChR aggregation. Skeletal muscle secretes muscle agrin (isoforms 3 and 6), which has no amino acid insertions at the Z site (agrin [Z0]) and is critical for the maintenance of the NMJ by binding to α-dystroglycan. LG, Laminin G-like domain; NtA, N-terminal agrin domain.

## Supplementary Table

**Supplementary Table 1. Clinical and genetic overview of CMS patients with *AGRN* mutations reported in the literature and in the present study**

| **Reference** | **Gender** | **Onset** | **Clinical forms:**  **Ocular/Facial/**  **Proximal/ Distal/Respiratory** | **CK** | **Accompanying performances** | **Anti-AchR anti-MuSK antibodies** | **RNS**  **(3 Hz)** | **Response to Treatment**  **AchEI/ 3,4-DAP/**  **β2-receptor agonist** | **Mutations** | **Mutation mode** |
| --- | --- | --- | --- | --- | --- | --- | --- | --- | --- | --- |
| Ref [[10](#_ENREF_10)] | F | Early childhood | +/+/+/+/ND | ND | thoracostenosis  weak pelvis | ND | + | -/ -/ + | G1709R | homozygous |
| Ref [[10](#_ENREF_10)] | M | Early childhood | +/ND/+/ND/ND | ND | thoracostenosis | ND | + | -/ +/ + | G1709R | homozygous |
| Ref [[7](#_ENREF_7)] | M | Early childhood | +/+/+/+/+ | ND | high-arched palate | - | + | +/ -/ - | Q353*  V1727F | compound heterozygous |
| Ref [[6](#_ENREF_6)] | F | 15 y | -/-/+/+/ND | N | joint hyperextension | - | + | -/ ND/ ND | G76S  Chr1del | compound heterozygous |
| Ref [[6](#_ENREF_6)] | M | 15 y | -/-/-/+/ND | N | - | - | + | -/ ND/ ND | G76S  Chr1del | compound heterozygous |
| Ref [[6](#_ENREF_6)] | M | 2 y | -/-/+/+/- | N | - |  | + | -/ -/ + | N105I  S455Qfs*8 | heterozygous |
| Ref [[6](#_ENREF_6)] | F | At birth | -/+/ND/+/- | N | - | - | + | -/ -/ + | N105I  S455Qfs*8 | heterozygous |
| Ref [[6](#_ENREF_6)] | M | 5 y | +/ND/+/+/ND | N | - | ND | + | ND/ ND/ + | G1871R | homozygous |
| Ref [[8](#_ENREF_8)] | M | 6 y | -/-/+/+/- | ND | - | ND | + | -/ ND/ - | L1176P | homozygous |
| Ref [[8](#_ENREF_8)] | M | y | -/-/+/+/- | ND | - | ND | + | -/ ND/ + | R1698C | homozygous |
| Ref [[11](#_ENREF_11)] | M | 21y | -/-/+/+/- | N | - | - | + | +*/ ND/ + | A1768P | homozygous |
| Ref [5] | F | 7y | -/-/+/+/- | N | - | ND | ND | +*/ ND/ + | A1768P | homozygous |
| Ref [5] | F | ND | ND | ND | ND | ND | ND | +*/ ND/ + | A1768P | homozygous |
| Ref [6] | M | 17mo | +/ND/+/-/ND | N  80U/L | - | - | + | +/ ND/ + | G1675S | homozygous |
| Present study | F | At birth | +/-/+/+/- | N118.9U/L | high-arched palate, enamel hypoplasia, scoliosis, ankle and carpal-joint hyper- extension | - | ND** | +/ ND/ ND | E42A  A1506T | compound heterozygous |

AChEI, acetylcholinesterase inhibitor; AchR, acetylcholine receptor; F, female; M, male; mo, months old; MUSK, muscle-specific tyrosine kinase; N, normal; ND, not determined; RNS, repeated nerve stimulation; y, years old; 3,4-DAP, 3,4-diaminopyridine

* Beneficial response during the first month, but then symptoms were aggravated.

** The patient did not cooperate during the RNS examination.

**Supplementary Table 2. The data of the patient’s motor nerve conduction***

| **Nerve** | **Latency (ms)** | **Amplitude (μV)** | **Conduction Velocity (m/sec)** |
| --- | --- | --- | --- |
| Left ulnar (wrist) - ADM | 4.13↑ | 0.29 ↓ |  |
| Left ulnar (elbow) - wrist | 7.38 | 0.15 | 35.4↓ |
| Right ulnar (wrist) - ADM | 4.21↑ | 0.12 ↓ |  |
| Right ulnar (elbow) - wrist | 6.06 | 0.24 | 64.9 |
| Left median (wrist) - APB | 3.62 | 0.15 ↓ |  |
| Left median (elbow) - wrist | 7.59 | 0.11 | 34.0 ↓ |
| Right median (wrist) - APB | 3.28 | 0.18 ↓ |  |
| Right median (elbow) - wrist | 6.23 | 0.16 | 47.5 ↓ |
| Left tibial (ankle) - AH | 3.43 | 0.89↓ |  |
| Left tibial (knee) - AH | 8.19 | 0.15 |  |
| Left tibial (knee) - ankle | 8.19 | 0.15 | 45.2 |
| Right tibial (ankle) - AH | 5.38↑ | 0.13↓ |  |
| Right tibial (knee) - AH | 9.98 | 0.29 |  |
| Right tibial (knee) - ankle | 9.98 | 0.29 | 45.7 |
| Left deep peroneal (ankle) - EDB | 2.42 | 0.043↓ |  |
| Left deep peroneal (fibular head) - EDB | 6.56 | 0.079 |  |
| Left deep peroneal (fibular head) - ankle | 6,56 | 0.079 | 48.3 |

ADM, abductor digiti minimi; AH, abductor hallucis; APB, Abductor Pollicis Brevis; EDB, Extensor digitorum brevis

* Electromyography of the right deep peroneal nerve was not performed due to an indwelling venous needle on the back of the right foot.

**Supplementary Table 3. The data of the patient’s sensory nerve conduction** *

| **Nerve** | **Peak Latency (ms)** | **Amplitude (μV)** | **Conduction Velocity (m/sec)** |
| --- | --- | --- | --- |
| Left ulnar, digit IV - wrist | 4.68↑ | 2.8 ↓ | 15.0 ↓ |
| Right ulnar, digit IV - wrist | 4.46↑ | 0.46 ↓ | 14.6 ↓ |
| Left median, palm - wrist | 4.97 ↑ | 3.3 ↓ | 19.1↓ |
| Right median, palm-wrist | 4.46 ↑ | 2.0↓ | 20.2↓ |
| Left superficial peroneal | 3.06 ↑ | 4.5 ↓ | 31.0 ↓ |
| Left Sural, | 4.87↑ | 3.6 ↓ | 12.3↓ |
| Right Sural | 2.59 ↑ | 5.1 ↓ | 23.2 ↓ |

* Electromyography of the right superficial peroneal nerve was not performed due to an indwelling venous needle on the back of the right foot.

**Supplementary Table 4. The data of the patient’s H-reflex**

| **Nerve** | **M-Latency**  **(ms)** | **H-Latency**  **(ms)** | **H-Amplitude**  **(mV)** | **H/M** |
| --- | --- | --- | --- | --- |
| Left tiblal | 5.0 | - | absence | - |
| Right tiblal | 5.3 | - | absence | - |

**Supplementary Table 5. The data of the patient’s motor unit potential (MUP)** *

| **Nerve** | **Mean Amplitude (mV)** | **Mean Duration (ms)** | **% Poly** |
| --- | --- | --- | --- |
| Left extensor digitorum muscle | 502 | 14.2↑ | 16.7 |
| Right tibialis anterior muscle | 1205 | 14.4↑ | 30.0 |

* The data of MUP of left tibialis anterior muscle and right extensor digitorum muscle were absent due to the patient’s uncooperation.
