# Supplementary figures and images for: Novel NtA and LG1 Mutations in Agrin in a Single Patient Causes Congenital Myasthenic Syndrome
Source: Front Neurol. 2020 Apr 9;11:239. doi: 10.3389/fneur.2020.00239 (PMC7160337; doi:10.3389/fneur.2020.00239)

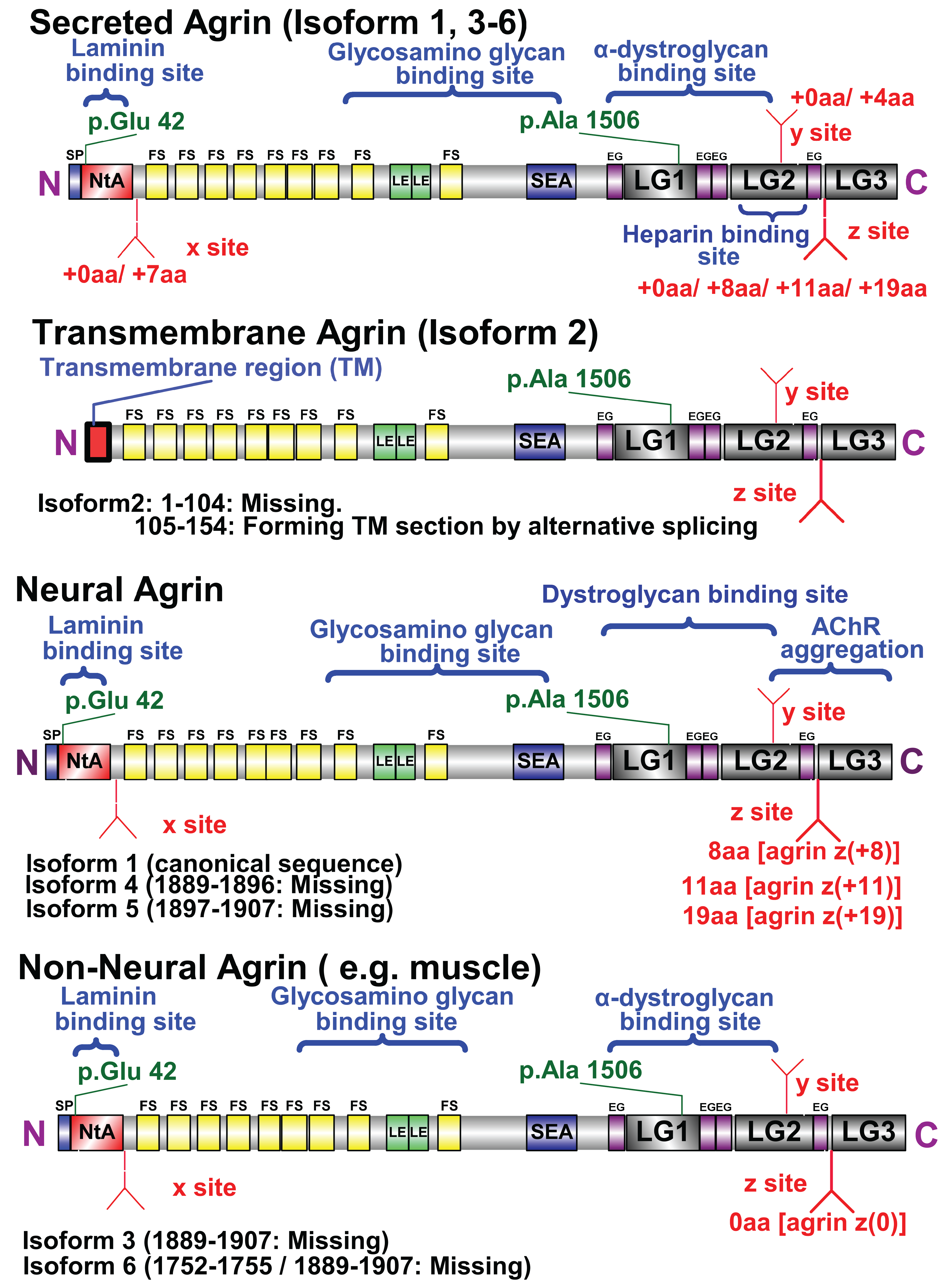

Supplement: Supplementary file 2 [file Image_1.TIF]
